# The Insertion of an Evolutionary Lost Four-Amino-Acid Cytoplasmic Tail Peptide into a Syncytin-1 Vaccine Increases T- and B-Cell Responses in Mice

**DOI:** 10.3390/v15081686

**Published:** 2023-08-03

**Authors:** Isabella Skandorff, Jasmin Gille, Emeline Ragonnaud, Anne-Marie Andersson, Silke Schrödel, Christian Thirion, Ralf Wagner, Peter Johannes Holst

**Affiliations:** 1 Department of Immunology and Microbiology, University of Copenhagen, Blegdamsvej 3B, 2200 Copenhagen, Denmark; isa@inprother.com; 2 InProTher, COBIS, Ole Maaloesvej 3, 2200 Copenhagen, Denmark; era@inprother.com (E.R.); aca@inprother.com (A.-M.A.); 3 Institute of Medical Microbiology and Hygiene, Molecular Microbiology, University of Regensburg Germany, 93053 Regensburg, Germany; jasmin.gille@klinik.uni-regensburg.de (J.G.); ralf.wagner@klinik.uni-regensburg.de (R.W.); 4 Department of Biomedical Sciences, University of Copenhagen, Blegdamsvej 3B, 2200 Copenhagen, Denmark; 5 Sirion Biotech GmbH, Am Haag 6, 82166 Graefelfing, Germany; schroedel@sirion-biotech.de (S.S.); thirion@sirion-biotech.de (C.T.)

**Keywords:** adenoviral vector, cell fusion, human endogenous retrovirus type W (HERV-W), R-peptide, Syncytin-1

## Abstract

Human endogenous retrovirus type W (HERV-W) is expressed in various cancers. We previously developed an adenovirus-vectored cancer vaccine targeting HERV-W by encoding an assembled HERV-W group-specific antigen sequence and the HERV-W envelope sequence Syncytin-1. Syncytin-1 is constitutively fusogenic and forms large multinucleated cell fusions when overexpressed. Consequently, immunising humans with a vaccine encoding Syncytin-1 can lead to the formation of extensive syncytia, which is undesirable and poses a potential safety issue. Here, we show experiments in cell lines that restoring an evolutionary lost cleavage site of the fusion inhibitory R-peptide of Syncytin-1 inhibit cell fusion. Interestingly, this modification of the HERV-W vaccine’s fusogenicity increased the expression of the vaccine antigens in vitro. It also enhanced Syncytin-1-specific antibody responses and CD8^+^-mediated T-cell responses compared to the wildtype vaccine in vaccinated mice, with a notable enhancement in responses to subdominant T-cell epitopes but equal responses to dominant epitopes and similar rates of survival following a tumour challenge. The impairment of cell–cell fusion and the enhanced immunogenicity profile of this HERV-W vaccine strengthens the prospects of obtaining a meaningful immune response against HERV-W in patients with HERV-W-overexpressing cancers.

## 1. Introduction

Human endogenous retrovirus (HERV) genes originate from now-extinct exogenous retroviruses that integrated into the genome of our ancestors. HERV type W (HERV-W) proviruses were acquired approximately 30–40 million years ago; subsequently, HERV-W genes spread throughout the genome via reinfections and retro-transpositions [1]. Since then, accumulations of mutations and truncations have compromised the coding capacity of most HERV genes, including HERV-W [1,2]. However, some HERV genes are still coding-competent and express functional proteins. For example, the *ERVWE1* provirus encodes the envelope (Env) protein Syncytin-1, which has been co-opted to play crucial roles in placentation [3,4,5]. Syncytin-1 facilitates the fusion of cytotrophoblasts, leading to the formation of the placental syncytiotrophoblast cell layer [3,4,6]; furthermore, Syncytin-1 is believed to be involved in maintaining foeto-maternal tolerance [7,8]. The expression of Syncytin-1 is strictly regulated by epigenetic mechanisms [9,10], and the aberrant expression of Syncytin-1 is associated with various pathologies, among others, cancer [11,12]. There is a growing number of reports on the expression of Syncytin-1 and gene products of other HERV-W loci in human cancer cells and tissues at either mRNA or protein levels (reviewed in [11,13,14,15,16]). The implication of HERV-W/Syncytin-1 expression in cancer is not fully understood, but the expression of HERV-W in cancer makes these antigens potential targets for cancer immunotherapy. 

In a recent study, we aimed to make a HERV-W-targeting cancer vaccine based on the virus-like-vaccine concept [17]. In a replication-deficient adenovirus vector, an assembled HERV-W group-specific antigen (Gag) sequence and the HERV-W Env sequence of Syncytin-1 were encoded [18]. We evaluated the vaccine-induced immune responses and anti-cancer efficacy in inbred mice, and we found that the HERV-W vaccine elicited both T-cell responses to different domains of the Env protein and the antibody recognition of the native form of Syncytin-1, which was expressed on the surfaces of the mouse cancer cells [18]. 

The HERV-W Env Syncytin-1 is, however, an atypical vaccine antigen as it is constitutively fusion-competent. When expressed on a cell surface, Syncytin-1 can induce cell–cell fusion via interaction with either of the identified receptors, human ASCT1 and ASCT2, and when Syncytin-1 is overexpressed, these interactions can form large, multinucleated cells. The immunisation of humans with a vaccine encoding the constitutively fusogenic Syncytin-1 would not only result in an immune attack of the HERV-W vaccine-infected cells but also adjacently fused non-transduced cells, worsening the local vaccine-induced tissue damage. To avoid this effect, we were interested in developing an immunogenic but non-fusogenic HERV-W vaccine. 

In the functional characterisation of Syncytin-1, mutagenesis studies found that Syncytin-1 fusion can be abolished by different mutations in both the extracellular and intracellular parts of Syncytin-1 while still enabling its surface expression [19,20,21]. One study showed that the substitution of cysteine with alanine at the third cysteine in a CX_6_CC disulfide motif, which is located in the extracellular part of the transmembrane subunit (TU) of Syncytin-1, impaired disulfide binding to a CXXC motif in its surface subunit (SU). This mutation inhibited cell–cell fusion but maintained Syncytin-1 surface expression [20]. Another study compared the cytoplasmic tail (CT) of Syncytin-1 with paralogous and orthologous HERV-W Env sequences from humans and different apes. It revealed that Syncytin-1 is constitutively fusogenic because it has lost the four amino acids that originally constituted the cleavage site for the fusion-inhibitory R-peptide in the CT [21]. When re-introducing a four-amino-acid-long consensus sequence, LQMV, Syncytin-1 was still surface-expressed, but the cell fusion activity was inhibited. This is because the human genome no longer holds any functional proteases from the HERV-W Pro-Pol loci. Thus, the LQMV-containing Syncytin-1 mutant is locked in a non-fusogenic conformation [21,22].

In the present study, we aimed to construct a non-fusogenic HERV-W vaccine without compromising the immunogenicity of the vaccine. We tested CX_6_CC motif-mutated and LQMV-reconstituted Syncytin-1 Env proteins as possible non-fusogenic HERV-W Env candidates in our vaccine constructs, and we explored the fusion capacity and surface expression of these constructs in human cell lines. Furthermore, we encoded the LQMV mutant in an adenovirus-vectored vaccine and examined its immunogenicity compared to the wt HERV-W vaccine in mice [18]. We show that the non-fusogenic LQMV mutant vaccine increased the cell surface expression of HERV-W Env in both human and murine cells. Additionally, this vaccine increased higher CD8^+^ T-cell responses towards subdominant antigens and increased antibody responses towards cancer cells expressing the native Syncytin-1 protein compared to the corresponding fusion-competent vaccine. The fusogenic and non-fusogenic HERV-W vaccines increased the survival of tumour-challenged mice to the same extent, possibly reflecting similar dominant epitope-specific responses. Thus, these results imply that we can avoid vaccine-induced cell–cell fusions while obtaining quantitatively higher antigen-specific immune responses by inserting LQMV into the CT of Syncytin-1. 

## 2. Materials and Methods

### 2.1. Cell Lines

The human T24 urinary bladder carcinoma cell line (HTB-4; ATCC, Manassas, VA, USA) and the human HEK293 epithelial kidney cell line (CRL-1573; ATCC, Manassas, VA, USA) were cultured in DMEM GlutaMAX. The murine RenCa renal cortical adenocarcinoma cell line from a male BALB/c mouse (CRL-2947, ATCC, Manassas, VA, USA) and the human A549 lung carcinoma cell line (CCL-185, ATCC, Manassas, VA, USA) were cultured in RPMI 1640 GlutaMAX and Ham’s F12 Nutrient Mix GlutaMAX media (31765035, Thermo Scientific™, Waltham, MA, USA), respectively. All media were supplemented with 10% FBS, 100 units/mL of penicillin–streptomycin (pen/strep) (15140122; Thermo Scientific™, Waltham, MA, USA), and 1 mM sodium pyruvate (11360070; Thermo Scientific™, Waltham, MA, USA). All cells were maintained at 37 °C and 5% CO_2_.

### 2.2. Antigen and Viral Vector Design

The sequences of HERV-W Gag and the wt HERV-W Env, Syncytin-1, were described in our recent study [18]. The HERV-W Gag sequence was obtained from an assembled sequence derived from the viral particles of the multiple sclerosis-associated retrovirus of the HERV-W family in a study by Komurian-Pradel et al. [23]. Based on the virus-like-vaccine principle, we included the assembled HERV-W Gag sequence in the vaccine construct to obtain in situ-formed HERV-W Gag particles presenting the HERV-W Env Syncytin-1 in a highly immunogenic fashion [17]. In the present study, three HERV-W vaccine plasmids were constructed: one encoding the wt Syncytin-1 and Gag (HERV-W_WT_), one encoding Syncytin-1 with four amino acids inserted into the CT region and HERV-W Gag (HERV-W_LQMV_), and one encoding Syncytin-1 with a cysteine-to-alanine exchange in the ectodomain and HERV-W Gag (HERV-W_C>A_).

The Syncytin-1 sequence, incl. the above-mentioned mutated variants in bold and/or underlined, is as follows: MALPYHIFLFTVLLPSFTLTAPPPCRCMTSSSPYQEFLWRMQRPGNIDAPSYRSLSKGTPTFTAHTHMPRNCYHSATLCMHANTHYWTGKMINPSCPGGLGVTVCWTYFTQTGMSDGGGVQDQAREKHVKEVISQLTRVHGTSSPYKGLDLSKLHETLRTHTRLVSLFNTTLTGLHEVSAQNPTNCWICLPLNFRPYVSIPVPEQWNNFSTEINTTSVLVGPLVSNLEITHTSNLTCVKFSNTTYTTNSQCIRWVTPPTQIVCLPSGIFFVCGTSAYRCLNGSSESMCFLSFLVPPMTIYTEQDLYSYVISKPRNKRVPILPFVIGAGVLGALGTGIGGITTSTQFYYKLSQELNGDMERVADSLVTLQDQLNSLAAVVLQNRRALDLLTAERGGTCLFLGEEC**C**YYVNQSGIVTEKVKEIRDRIQRRAEELRNTGPWGLLSQWMPWILPFLGPLAAIILLLLFGPCIFNLLVNFVSSRIEAVKLQMVLQMEPKMQSKTKIYRRPLDRPASPRSDVNDIKGTPPEEISAAQPLLRPNSAGSS*.

In all three plasmids, the sequences coding for the Env and Gag proteins were separated by a self-cleavable P2 A peptide sequence, and a CMV promoter controlled the antigen expression. A control plasmid for transfection encoded a copGFP sequence. GenScript Biotech (Piscataway, NJ, USA) synthesised all plasmids. 

The HERV-W_WT_ and HERV-W_LQMV_ constructs were further cloned into a shuttle vector in *E. coli* and hereafter into a BAC vector containing the backbone of a replication-deficient human adenoviral vector type 19a/64 (hAd19a/64) (lacking E1 and E3 genes) [24]. A negative control vaccine (Neg. ctrl vaccine) contained the same vector but did not encode any antigens. 

### 2.3. Adenoviral Vector Production

The hAd19a/64 vaccines were produced by Sirion Biotech, following the procedure described in [25]. In brief, after the cloning of the HERV-W antigens into the hAd19a/64 backbone in BAC cells, the DNA was purified, linearized, and then transfected into a modified HEK293 production cell line. In the HEK293 cells, the viral constructs were amplified to a large-scale lysate, and from here, the viruses were purified. The purified viruses were tittered in parallel via the immunohistochemical staining of the adenoviral hexon protein. Virus-derived DNA was isolated and sequenced for quality control. 

### 2.4. Surface Expression of Transfected HEK293 and T24 Cells and Transduced A549 Cells

HEK293 and T24 cells were transfected with either HERV-W_WT_, HERV-W_LQMV_, or HERV-W_C>A_ plasmids using PEI and Opti-MEM (11058021; Thermo Scientific™, Waltham, MA, USA) in complete DMEM media without pen/strep. Transfection with PEI and Opti-MEM was carried out in the ratios of DNA to PEI, 1:3, and DNA to Opti-MEM, 1:100, meaning 3 µg of DNA to 9 µL (1 mg/mL) of PEI to 300 µL of Opti-MEM. Each condition was performed in duplicates. The cells were incubated for 24 h prior to cell surface staining. 

Human A549 cells were transduced with the hAd19a/64 HERV-W_WT_, HERV-W_LQMV_, or an empty vaccine (Neg. ctrl vaccine) at a multiplicity of infection (MOI) of 10. Each condition was performed in triplicate, and the cells were stained after 24 h of incubation. 

The following antibody staining of the transfected and transduced cells was carried out in a FACS buffer consisting of PBS with 1% BSA and 0.1% NaN_3_. For the cell surface staining of the HERV-W Env surface subunit, the cells were incubated with 15 µg/mL of primary rabbit anti-human HERV polyclonal antibody (PA5-22819; Invitrogen™, Waltham, MA, USA) for 1 h at 4 °C. Following this, the cells were stained with secondary PE donkey anti-rabbit IgG antibody (406421; BioLegend^®^, San Diego, CA, USA; 1:100) and eBioscience™ Fixable Viability Dye eFlour™ 780 (65-0865; Invitrogen™, Waltham, MA, USA; 1:1000) for 30 min at 4 °C. Next, the cells were fixated in 1% paraformaldehyde (PFA) for 15 min at 4 °C, and flow cytometry was performed using either the LSRFortessa™ 3-laser or 5-laser cell analyser (BD Biosciences, Franklin Lakes, NJ, USA). The flow cytometry data were analysed with FlowJo™ v10 analysis software and GraphPad Prism 9. 

### 2.5. Visualisation of Cells Using Transmission Electron Microscopy (TEM) and Light Microscopy

T24 and A549 cells were seeded on Thermanox coverslips (150067; Thermo Scientific™, Waltham, MA, USA) in 24-well plates and hereafter transfected or transduced as described above except in the case of the A549 cells, which were transduced with 50MOI. Prior to cell seeding, the coverslips for the A549 cells were pre-coated with poly-L-lysine. Following 24 h of incubation, the cells were fixed with 2% glutaraldehyde in a 0.05 M sodium phosphate buffer (pH 7.2). As a control, T24 cells were transfected with a plasmid encoding a copGFP sequence and incubated for 48 h. TEM was performed by the Core Facility for Integrated Microscopy at the University of Copenhagen (see detailed description in [18,25]).

Using a ZOE Cell Imager (BioRad, Hercules, CA, USA), light microscopy pictures of unstained and non-fixed T24 cells 24 h after transduction with 25MOI of the hAd19a/64 HERV-W_WT_, HERV-W_LQMV_, or Neg. ctrl vaccine were obtained.

### 2.6. Evaluation of Gag Expression via Western Blotting 

HERV-W Gag expression was evaluated via Western blotting, as described previously in [18]. In brief, A549 cells were lysed 24 h after transduction with 50MOI of either the HERV-W_WT_ vaccine or the HERV-W_LQMV_ vaccine. Denatured samples were run on a NuPAGE™ Bis-Tris Mini Gel (NP0321; Invitrogen™, Waltham, MA, USA) under reducing conditions and transferred to a nitrocellulose membrane (IB230002; Invitrogen™, Waltham, MA, USA). The membrane was incubated overnight at 4 °C with the primary antibodies: the anti-T2A-antibody (Crb200569d; CRB discoveries, Cleveland, UK; 1:2000) to detect the P2A peptide on HERV-W Gag and the housekeeping control protein anti-GAPDH antibody (ab181602; Abcam, Cambridge, UK; 1:8000). The bound primary antibody was detected after 1 h of incubation with the secondary polyclonal goat anti-rabbit IgG antibody (P0448; Dako, Glostrup, Denmark), using LumiGlo Chemiluminescent (5430; KPL, LGC group, Teddington, UK) or SuperSignal West Femto Maximum Sensitivity Substrate (34095; Thermo Scientific™, Waltham, MA, USA). The relative expression differences in the protein bands were analysed with iBright analysis software, V5.1.0. 

### 2.7. Maturation, Transduction, and Staining of Murine Bone Marrow-Derived Dendritic Cells (BMDCs) and Measurement of Pro-Inflammatory Biomarkers

BMDCs derived from the femurs of two BALB/c mice were isolated and matured based on a study published by Jin et al. [26]. Detailed descriptions of the maturation conditions, transduction, and staining are provided in [18]. Twenty-four hours after the transduction of the murine BMDCs, the supernatant was collected. The concentrations of four different proinflammatory biomarkers were determined using a customised V-PLEX mouse proinflammatory cytokine panel 1 kit (K15048D; Mesoscale, Rockville, MD, USA). The supernatants were diluted 1:5, and the samples were assessed in duplicates. The biomarker concentrations were analysed with a MESO QuickPlex SQ 120 MM instrument (Mesoscale, Rockville, MD, USA). 

### 2.8. Animal Procedures and Serum Isolation

Female BALB/c mice from Envigo were obtained at 6–8 weeks of age and housed at the Panum Institute, University of Copenhagen. The mice were acclimatised for at least one week prior to any experiments, and all the experiments were performed in accordance with the national guidelines. The experimental procedures were approved by the National Animal Experiments Inspectorate (Dyreforsøgstilsynet, license no. 2019-15-0201-00203). 

Prior to subcutaneous (s.c.) vaccinations, the mice were anaesthetised with isoflurane. The mice were vaccinated s.c. in the lower right limb with 30 µL of the relevant vaccine, which contained 2 × 10^7^ infectious units (IFU) diluted in 1 × PBS. At the end of each experiment, the mice were euthanised via cervical dislocation. 

Blood samples were collected prior to and on the last day of the vaccination studies. Serum was isolated from the blood samples via two centrifugations at 800× *g* for 8 min at 8 °C.

Intravenous (i.v.) tumour inoculation was performed as described previously in Skandorff et al. [18]. Each mouse was challenged with 0.5 × 10^6^ RenCa cells modified to express the HERV-W Env Syncytin-1 (see previous study [18]). These cells were diluted in 1 × PBS, and 100 µL of cell suspension was i.v. injected into the tail vein. The mice were randomised, and four days after the tumour challenge, the mice were divided into three groups; each group was vaccinated with either 2 × 10^7^ IFU of the HERV-W_WT_ vaccine (*n* = 15), HERV-W_LQMV_ vaccine (*n* = 15), or the Neg. ctrl vaccine (*n* = 10). The mice were continuously monitored, and when humane endpoints were reached (starey coat, bent over position, or reduced mobility) the mice were euthanised via cervical dislocation. The lungs were collected and evaluated for the presence of tumour nodules. One mouse in the HERV-W_WT_ vaccinated group and two in the HERV-W_LQMV_ group showed no signs of sickness throughout the experiment. When evaluating the presence of tumour nodules in the lungs of these remaining mice, the HERV-W_WT_-vaccinated mouse showed one minor nodule. In contrast, no nodules were visible in the HERV-W_LQMV_-vaccinated mice. The probability of survival was calculated between groups two-and-two using a log-rank Mantel-Cox test in GraphPad Prism 9 with a statistical significance level defined as * = *p* < 0.05. 

### 2.9. Evaluation of HERV-W Env-Specific Antibody Responses

Antibodies, from serum isolated from BALB/c mice prior to and at the end of the vaccination, were evaluated via flow cytometry for binding to RenCa cells modified to stably express HERV-W Env. Pre- and end-bleed sera were diluted 1:20 and added to the HERV-W Env^+^ Renca cells for 1 h at 4 °C. Following this, the cells were stained with PE goat anti-mouse IgG (405307; BioLegend^®^, San Diego, CA, USA; 1:100) and eBioscience™ Fixable Viability Dye eFlour™ 780 (65-0865; Invitrogen™, Waltham, MA, USA; 1:1000) for 30 min at 4 °C. Finally, the cells were fixated in 1% PFA, and flow cytometry was performed on an LSRFortessa™ 3-laser flow cytometer (BD Biosciences, Franklin Lakes, NJ, USA). FlowJo™ v10 and GraphPad Prism 9 were used to analyse the antibody responses. Statistical significance levels were calculated using the Mann–Whitney *t*-test (* = *p* < 0.05 and ** = *p* < 0.01). 

### 2.10. Evaluation of HERV-W Gag and Env T-Cell Responses 

**Figure 3:**
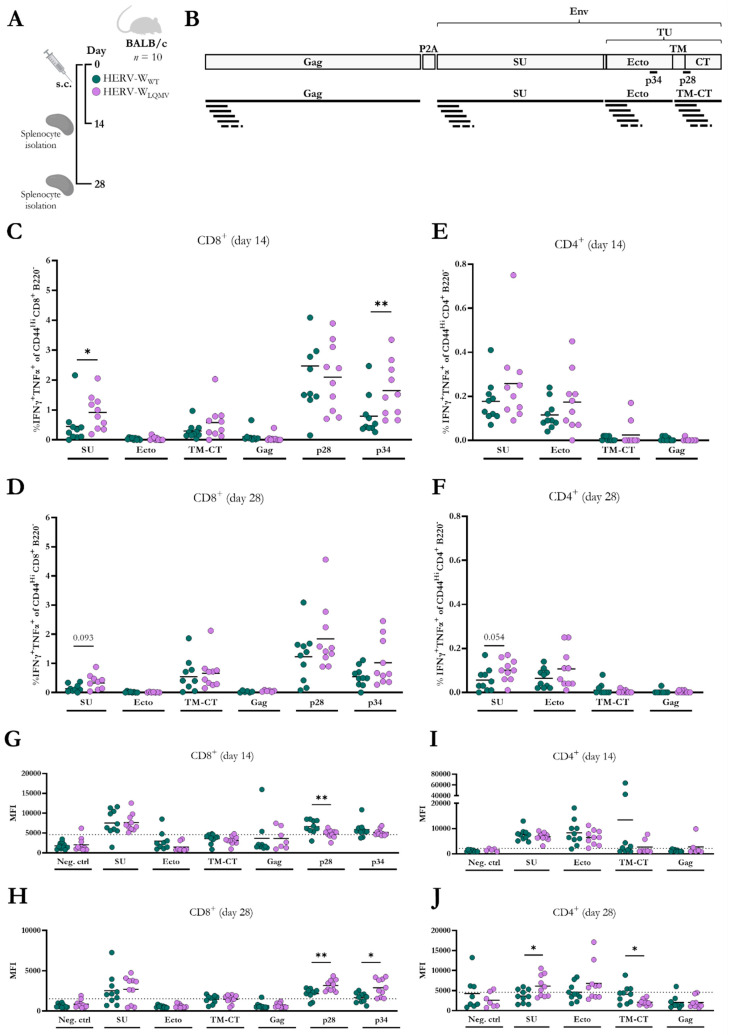
CD8^+^ and CD4^+^ T-cell responses in BALB/c mice vaccinated with the two HERV-W vaccines. (**A**) Schematic representation of the immunisation study with the HERV-W_WT_ and HERV-W_LQMV_ vaccines. BALB/c mice were immunised subcutaneously (s.c.) in the lower limb on day zero, and splenocytes were harvested either 14 days or 28 days later. For each of the two experiments, each vaccine group included ten mice. (**B**) Illustration of the vaccine-encoded antigens, visualising the four different 16-mer peptide pools spanning HERV-W Gag, HERV-W Env SU, or the two HERV-W TU regions: the ectodomain (Ecto) or the TM-CT. The figure also illustrates the origin of the two 9-mer peptides: peptide 28 (p28, TM-CT) and peptide 34 (p34, Ecto). Graphs show the percentages of IFNγ- and TNFα-expressing CD8^+^ T-cells 14 (**C**) and 28 (**D**) days after immunisation or CD4^+^ T-cells 14 (**E**) and 28 (**F**) days after immunisation. Geometric mean fluorescent intensity (MFI) of IFNγ-expressing CD8^+^ T-cells on day 14 (**G**) and day 28 (**H**), as well as CD4^+^ T-cells on day 14 (**I**) and day 28 (**J**) after vaccination. The gating strategy is illustrated in supplementary Figure S3A. The individual bullet depicts the response from one mouse, and the horizontal line indicates the mean of the group. Statistically significant differences (non-parametric, two-tailed Mann–Whitney test) between T-cell responses are marked by * = *p* < 0.05 and ** = *p* < 0.01. The dotted lines indicate a lower limit for a positive response, and they are based on the mean plus two times the standard deviation of the mean of the responses for T-cells without peptide stimulation (Neg. ctrl).

T-cell responses were evaluated using splenocytes isolated from the spleens of the vaccinated mice, as described previously [18]. The splenocytes were stimulated for 5 h with pools of 16-mer peptides, which overlapped by 11 amino acid and that together spanned either the HERV-W Env Syncytin-1 cell surface subunit, the ectodomain of Syncytin-1’s transmembrane subunit, the Syncytin-1 transmembrane domain and the cytoplasmic tail of the transmembrane subunit, or the assembled HERV-W Gag (see Figure 3B). The splenocytes were also stimulated with two previously identified 9-mer peptides: peptide 28 (p28, FGPCIFNLL), which originated from the overlapping sequence between the transmembrane domain and the cytoplasmic tail of the transmembrane subunit of Syncytin-1, and peptide 34 (p34, CYYVNQSGI), which originated from the ectodomain of the transmembrane subunit of Syncytin-1 [18]. 

Following peptide stimulation, the splenocytes were stained with the following cell surface fluorophore-conjugated antibodies: BV421 rat anti-mouse CD8b antibody (126629; BioLegend^®^, San Diego, CA, USA), PE-Cy7 rat anti-mouse CD4 (561099; BD Biosciences, Franklin Lakes, NJ, USA), PerCP-Cy5.5 rat anti-mouse CD45R/B220 (552771; BD Biosciences, Franklin Lakes, NJ, USA), and FITC rat anti-mouse CD44 (553133; BD Biosciences, Franklin Lakes, NJ, USA). The splenocytes were fixated in 1% PFA and permeabilised with saponin before they were stained with two intracellular fluorophore-conjugated antibodies: APC rat anti-mouse IFNγ (554413; BD Biosciences, Franklin Lakes, NJ, USA) and PE rat anti-mouse TNFα (554419; BD Biosciences, Franklin Lakes, NJ, USA). Finally, flow cytometry was carried out on an LSRFortessa-3 flow cytometer (BD Biosciences, Franklin Lakes, NJ, USA), and the data were analysed using FlowJo™ v10 and GraphPad Prism 9. 

The percentage of the responses measured in the unstimulated samples for each mouse (background) was subtracted from the percentage of responses measured in the stimulated samples of the corresponding mouse. As a lower limit of positive geometric mean fluorescent intensity (MFI) IFNγ T-cell responses, the minimum response was defined as a response above the mean plus two times the standard deviation of the MFI of the splenocytes that were not stimulated with peptides (Neg. ctrl) (as seen in Figure 3G–J). However, when calculating response differences between the two vaccine groups, all responses were included. Response differences were calculated using the non-parametric Mann–Whitney test, with statistical significance levels defined as * = *p* < 0.05 and ** = *p* < 0.01. Values below zero were excluded from the graphs. 

## 3. Results

### 3.1. Characterisation of Non-Fusogenic HERV-W Envs 

**Figure 1:**
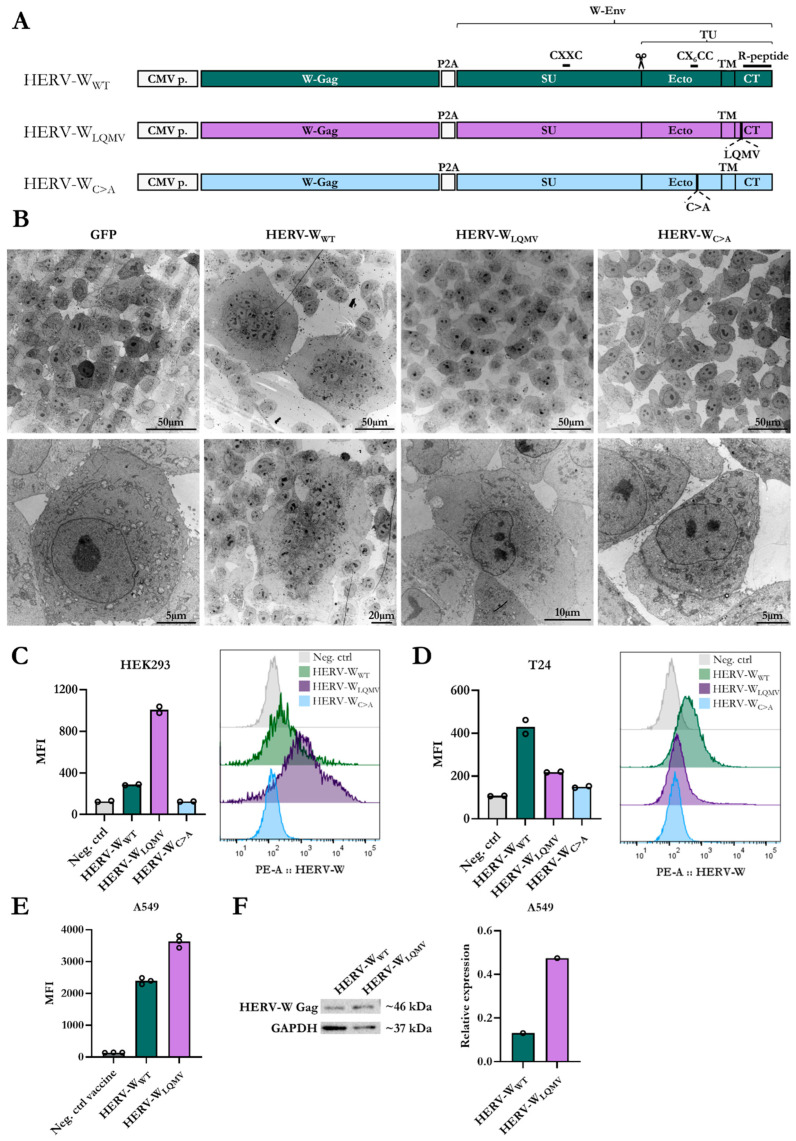
Characterisation of non-fusogenic mutants of HERV-W Env. (**A**) Schematic overview of plasmids encoding an assembled HERV-W Gag (W-Gag) sequence and three different versions of the HERV-W envelope (W-Env), Syncytin-1, sequences: wildtype Env (green, HERV-W_WT_), Env with LQMV insertion (purple, HERV-W_LQMV_), and Env with a C>A amino acid substitution (blue, HERV-W_C>A_). The Gag and Env antigens are separated by a self-cleavable P2A peptide sequence, and the expression of the antigens is controlled by a CMV promoter (CMV p.). The illustration also depicts the location of the R-peptide, the CX_6_CC and CXXC disulfide motifs, and the different domains of the Env: the surface subunit (SU) and the transmembrane subunit (TU), where the TU contains the ectodomain (Ecto), the transmembrane domain (TM), and the cytoplasmic tail (CT). The scissor illustrates the cleavage site between SU and TU. (**B**) Transmission electron microscopy (TEM) pictures of human T24 cells transfected with plasmids encoding GFP, HERV-W_WT_, HERV-W_LQMV_, or HERV-W_C>A_. The top row shows an overview of several cells, and the bottom row shows individual cells at a higher magnification. (**C**) (**right**) Graph showing the geometric mean fluorescent intensity (MFI) of the HERV-W Env surface expression of two technical repeats of transfected HEK293 cells, as indicated by bullets, and (**left**) histograms showing the distribution of one representative sample of each of the HERV-W plasmids and a negative control without transfection (Neg. ctrl). (**D**) The same as (**C**) but depicting results for T24 cells. (**E**) The MFI of the HERV-W Env surface expression of A549 cells 24 h after transduction with the hAd19a/64 vaccines encoding either HERV-W_WT_, HERV-W_LQMV_, or the empty expression cassette (Neg. ctrl vaccine). Bullets illustrate the three technical replicates per condition. (**F**) The Western blot shows HERV-W Gag expression in A549 cells 24 h after transduction with either the HERV-W_WT_ or HERV-W_LQMV_ vaccine. The right graph shows the difference in the expression of the HERV-W Gag in the Western blot between the two vaccines, relative to the expression of the housekeeping protein GAPDH.

To generate a non-fusogenic Syncytin-1 antigen, we first considered the evolutionary adaptation that rendered Syncytin-1 constitutively fusogenic. The C-terminal R-peptide of gamma-retroviruses inhibits Env fusion activity, but in the domestication of the HERV-W Env Syncytin-1, this protein acquired a four-amino-acid deletion mutation in the R-peptide cleavage site, making Syncytin-1 constitutively fusogenic. Based on human HERV-W Env paralogous sequences, Bonnaud et al. deduced a four-amino-acid sequence, LQMV [21]. Thus, in our first mutant HERV-W vaccine, we inserted the “lost” fusion-inhibitory R-peptide cleavage site into the Env CT (amino acids LQMV) and encoded this Env sequence in a plasmid together with an assembled sequence of HERV-W Gag (HERV-W_LQMV_) (Figure 1A). Other functional and structural studies of Syncytin-1 have shown that certain insertion and deletion mutations can maintain Env surface expression while abolishing fusion activity [19,20]. For our second HERV-W mutant vaccine, we exchanged the last cysteine in the CX_6_CC motif of the Env TU ectodomain with alanine (CX_6_CC → CX_6_CA), which was previously shown to abrogate the disulfide bond between the TU and SU [20]. Again, we encoded this Env into a plasmid together with HERV-W Gag (HERV-W_C>A_) (Figure 1A). The expression of the antigens was controlled using a CMV promoter, and the Gag and Env sequences were separated using a self-cleavable P2A peptide sequence. 

We assessed the cell morphology of the transfected human T24 cells via transmission electron microscopy (TEM). T24 cells are highly fusion-permissive when expressing Syncytin-1, which results in large, multinuclear cells, as seen when transfected with plasmids containing the wt Syncytin-1 together with the assembled HERV-W Gag sequence (HERV-W_WT_) (Figure 1B, Supplementary Figure S1A). In contrast, the T24 cells transfected with plasmids of HERV-W_LQMV_ and HERV-W_C>A_ were mono-nuclear, like the GFP control (Figure 1B). 

We further assessed the surface expression of the Env SU region of the easily transfected human HEK293 (Figure 1C) and T24 (Figure 1D) cells via flow cytometry. Consistent with the original study by Cheynet et al., the surface expression of the Env of the HERV-W_C>A_ mutant was lower than for HERV-W_WT_ in the T24 cells and almost absent in the HEK293 cells [20] (Figure 1C,D). Oppositely, the geometric mean fluorescent intensity (MFI) of the surface expression of Env was higher for the HERV-W_LQMV_ mutant than the HERV-W_WT_ in transfected HEK293 cells (Figure 1C) but lower than for HERV-W_WT_ in transfected T24 cells (Figure 1D). The removal of the disulfide bond connecting the TU and SU of the Env of the HERV-W_C>A_ mutant possibly affects the structure of the surface-expressed Env via the introduction of novel antigen structures that do not mimic the native Syncytin-1 protein. Considering this, and that the HERV-W_C>A_ mutant’s level of the cell surface expression of Env was low, we decided to continue with the HERV-W_LQMV_ mutant for the generation of a virus-vectored HERV-W vaccine. 

To generate a vaccine candidate for in vivo testing, we produced replication-deficient human adenoviral vector type 19 a/64 (hAd19a/64 vector) vaccines encoding the antigens from the HERV-W_WT_ and HERV-W_LQMV_ plasmids (as seen in Figure 1A). Following the transduction of human A549 cells, we evaluated the surface expression levels of the Env protein via flow cytometry (Figure 1E; gating strategy in Supplementary Figure S1B) and the intracellular expression of the Gag protein Western blotting (Figure 1F), respectively. In line with the transfection experiments with HEK293 cells, the surface expression level of Env was higher for the A549 cells transduced with the HERV-W_LQMV_ vaccine than the HERV-W_WT_ vaccine (Figure 1E, Supplementary Figure S1C). Similarly, the Gag protein concentration was higher in the HERV-W_LQMV_ transduced cells than in the HERV-W_WT_ transduced cells (Figure 1F). By examining the transduced A549 cells using light microscopy, we could again confirm the presence of multinucleated cells among the HERV-W_WT_ transduced cells, but none of these were present in the cells transduced with HERV-W_LQMV_ or an empty vectored vaccine (Neg. ctrl vaccine) (Supplementary Figure S1D). 

In a recent study of the HERV-W_WT_ vaccine, we further explored whether encoding HERV-W Gag and Env could give rise to Env-covered extracellular virus-like particles (VLPs) [18]. However, in accordance with our previous study, we could not observe any extracellular VLPs via TEM with either the HERV-W_WT_ or HERV-W_LQMV_ vaccine (Supplementary Figure S1E). 

### 3.2. Effects of Syncytin-1 in Transduced Bone Marrow-Derived Dendritic Cells 

**Figure 2:**
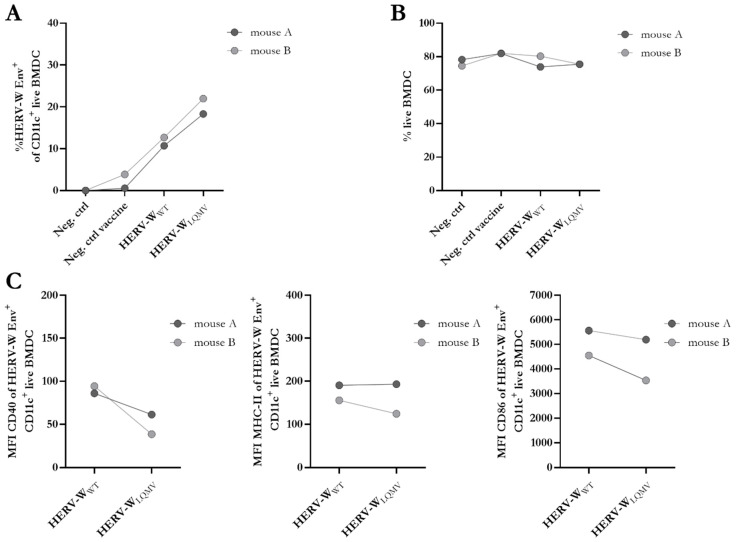
Evaluation of the effect of the non-fusogenic HERV-W_LQMV_ vaccine on activated BMDCs. (**A**) Percentage of surface expression of HERV-W Env of CD11^+^ live bone-marrow-derived dendritic cells (BMDCs) from two BALB/c mice, analysed via flow cytometry. (**B**) Percentage of live BMDCs. (**C**) Geometric mean fluorescent intensity (MFI) of the activation markers CD40 (**left**), MHC-II (**middle**), and CD86 (**right**) on the cell surfaces of the BMDCs expressing CD11 c and HERV-W Env. All bullets depict the mean of two technical repeats from each of the two mice.

Previously, we observed the association of vaccine immunogenicity with differences in the expression of activation markers on the surfaces of matured murine bone marrow-derived dendritic cells (BMDCs) [18,25]. In this study, we measured the surface expression of Syncytin-1 (from here on HERV-W Env) 24 h after transduction with either HERV-W_WT_ or HERV-W_LQMV_ and compared it to a lack of transduction (Neg. ctrl) or an empty vectored hAd19a/64 vaccine (Neg. ctrl vaccine) (Supplementary Figure S2A). Regarding its expression in human HEK293 with plasmids and the use of viruses in A549 cells, the Env cell surface expression on murine BMDCs was higher for HERV-W_LQMV_ than HERV-W_WT_ (Figure 2A), while the cell viability was similar (Figure 2B). No cell–cell fusions were observed with any of the vaccines; therefore, we did not anticipate any differences in the expression of the activation markers. Indeed, the percentages and MFI of HERV-W Env^+^ cells expressing MHC class II (MHC-II) and CD86 on their surfaces were similar between the two vaccines (Figure 2B, Supplementary Figure S2B). However, the MFI of CD40 on HERV-W Env^+^ cells appeared slightly lower for HERV-W_LQMV_ than HERV-W_WT_ (Figure 2C). We also measured the concentrations of four different secreted proinflammatory biomarkers in the supernatant 24 h after transduction but found no differences except for a modest increase in the concentration of TNFα with the HERV-W_LQMV_ vaccine when compared to HERV-W_WT_ and the Neg. ctrl vaccine (Supplementary Figure S2C). Overall, from these experiments, it is not possible to claim any clear differences in innate immunogenicity within the ex vivo-transduced BMDCs apart from the greater levels of cell surface Env expression of the BMDCs transduced with the HERV-W_LQMV_ vaccine.

### 3.3. The LQMV Mutant Vaccine Exhibit Increased T-Cell Immunogenicity 

To explore whether the LQMV mutant vaccine was equally immunogenic to the wt vaccine in vivo, we vaccinate BALB/c mice subcutaneously (s.c.) and evaluated their cellular and humoral immune responses after 14 days (the expected peak of T-cell responses) and 28 days (early memory responses) (Figure 3A). Using flow cytometry, we measured the IFNγ and TNFα responses of CD8^+^ and CD4^+^ T-cells via intracellular staining after peptide stimulation with two identified HERV-W Env-responding 9-mer peptides (peptide 28 and peptide 34), as well as three pools of 16-mer peptides overlapping by 11 amino acids (for the gating strategy, see Supplementary Figure S3A). The peptide pools were made to span three different sections of the Env protein: SU, the ectodomain of the TU, and the transmembrane (TM) and CT regions of the TU (Figure 3B), and these and the single peptides were identified and evaluated in our recent study [18]. In this present study, we also used a pool of overlapping 16-mer peptides spanning the HERV-W Gag protein sequence (Gag) to measure potential T-cell responses to HERV-W Gag (Figure 3B). 

Interestingly, the T-cell responses to the HERV-W_LQMV_ vaccine were generally higher than the responses to the HERV-W_WT_ vaccine. The percentage of IFNγ and TNFα CD8^+^ T-cell responses to peptide 34 from the TU ectodomain and the SU peptide pool were significantly higher for the HERV-W_LQMV_ vaccine group compared to the HERV-W_WT_ vaccine group on day 14 (Figure 3C). The same tendency was observed on day 28 (Figure 3D) and for IFNγ-producing CD8^+^ T-cell responses on day 14 (Supplementary Figure S3B), and with a reduced magnitude on day 28 (Supplementary Figure S3C). Consistent with our previous HERV-W vaccine study, the double-positive (IFNγ^+^TNFα^+^) and single-positive (IFNγ^+^) CD8^+^ T-cell responses elicited to peptide 28 were generally the highest of all the peptide conditions [18]. However, in this current study, the two HERV-W vaccine groups responded similarly to this major epitope. 

Furthermore, and in agreement with the previous study [18], vaccine-specific CD4^+^ T-cell responses were generally quite low in the BALB/c mice. However, we could still observe IFNγ^+^TNFα^+^-producing CD4^+^ T-cells responding to the peptide pools of the SU region and the ectodomain on day 14 and day 28; in most cases, these responses were the strongest for the HERV-W_LQMV_ vaccine group, but the difference between the groups did not achieve statistical significance (Figure 3E,F). The percentages of IFNγ^+^ CD4^+^ T-cell responses were almost indistinguishable on day 14 (Supplementary Figure S3D), but on day 28, the responses to the ectodomain were significantly higher for the HERV-W_LQMV_ group than for the HERV-W_WT_ group (Supplementary Figure S3E).

In contrast to all the other results, the MFI of IFNγ^+^ CD8^+^ T-cell responses to peptide 28 of the TM-CT region was higher for the HERV-W_WT_ vaccine group than the HERV-W_LQMV_ vaccine group on day 14 (Figure 3G), but then the opposite occurred on day 28 (Figure 3H). We speculate whether this observation can reflect a still-maturing immune response on day 14 which settled on day 28. At later stages of the response, both vaccine groups are expected to be at the resting stage, and we would imagine that a more immunogenic vaccine would provide both the quantitatively and qualitatively strongest responses, which is what we observed for the HERV-W_LQMV_ vaccine compared to the HERV-W_WT_ vaccine on day 28. 

The MFI of the IFNγ CD4^+^ T-cell responses on day 14 and day 28 were similar for most epitopes, but at 28 days, we observed a significantly higher MFI for the T-cells responding to the SU peptide pool in the HERV-W_LQMV_-immunised mice (Figure 3I,J). We oppositely observed a higher MFI for the TM-CT peptide pool in the HERV-W_WT_-immunised mice, but this was a mean responsiveness calculated from a very low-responding cell population (comparing Figure 3E,J). 

In this study, we also aimed to measure T-cell responses to HERV-W Gag by using a pool of 16-mer peptides that spanned the protein sequence. However, the responses to this region were very sparse and low. We only observed relevant responses in a few cases (Supplementary Figure S3F), but not to an extent to which we could observe differences between the two vaccine groups. 

Overall, we conclude that the HERV-W_LQMV_ vaccine increased T-cell responses, particularly for minor epitopes at early time points, and increased T-cell functionality with increasing consistency over time. 

### 3.4. HERV-W_LQMV_ Vaccine Induces Higher Antibody Responses but Similar Tumour Survival as the HERV-W_WT_ Vaccine 

**Figure 4:**
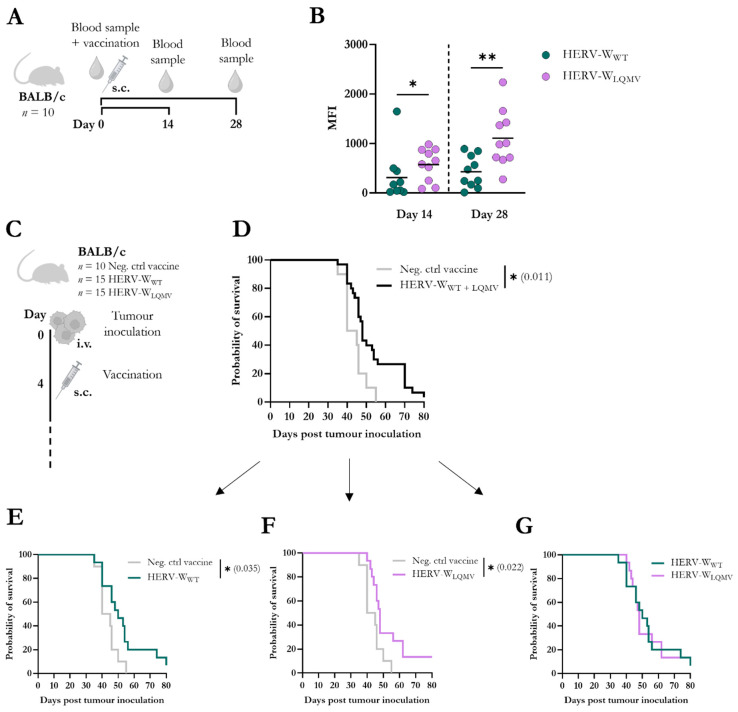
Evaluation of antibody responses and tumour efficacy in mice vaccinated with HERV-W_LQMV_. (**A**) Schematic representation of the vaccination study using BALB/c mice immunised with the HERV-W_WT_ or HERV-W_LQMV_ vaccine (*n* = 10 per group) (also illustrated in Figure 3A). Mice were vaccinated subcutaneously (s.c.) on day zero. Blood samples were taken prior to vaccination and at the end of the study, either on day 14 or day 28. (**B**) The graph shows the geometric mean fluorescent intensity (MFI) of serum IgG binding to the surface of HERV-W Env-expressing live RenCa cells, measured via flow cytometry. Each bullet represents one mouse, and the bold line shows the mean of the responses of the group. Response differences between the two vaccine groups were calculated using the Mann–Whitney test, and statistical significance is defined as: * = *p* < 0.05, ** = *p* < 0.01. (**C**) Overview of tumour study with HERV-W Env^+^ RenCa cells injected intravenously (i.v.) into the tail veins of BALB/c mice on day zero. On day 4, the mice were vaccinated s.c. with either of the two HERV-W vaccines or the empty control vaccine (Neg. ctrl vaccine). The mice were monitored until humane endpoints were reached. (**D**) Graph showing the survival of mice challenged with HERV-W Env^+^ RenCa tumours and immunised with the Neg. ctrl vaccine compared to the two HERV-W vaccines together. (**E**–**G**) Survival curves show the three vaccine groups compared two-and-two. The statistical survival difference between the groups was calculated using the log-rank test (Mantel–Cox), * = *p* < 0.05.

From the same mice that were used to measured T-cell responses, blood samples were also obtained prior to vaccination (pre-bleed) and on the day of the splenocyte isolation (end-bleed): day 14 or day 28 after vaccination (Figure 4A). We isolated serum, and via flow cytometry, we tested the binding of serum IgG to a murine RenCa cancer cell line that we had, in a previous study, modified to stably express the native HERV-W Env protein on the surface (Supplementary Figure S4A) [18]. The total serum IgG binding from HERV-W_LQMV_-vaccinated mice was significantly greater compared to the HERV-W_WT_ mice on day 14, and this difference was further increased on day 28 after vaccination (Figure 4B, Supplementary Figure S4B).

Since both T-cell and IgG responses were higher for the HERV-W_LQMV_-vaccinated mice than the HERV-W_WT_-vaccinated mice, we were curious whether this could also reflect improved tumour protection against HERV-W Env-expressing tumours. The BALB/c mice were injected intravenously (i.v.) in the tail with the HERV-W Env-expressing murine RenCa tumour cells. The mice were randomised, and four days after the tumour challenge, the mice were vaccinated with the HERV-W_WT_ (*n* = 15), the HERV-W_LQMV_ (*n* = 15), or the Neg. ctrl vaccine (*n* = 10) (Figure 4C). Together (Figure 4D) and individually (Figure 4E,F), the two HERV-W vaccines significantly increased the probability of survival compared to the control group. However, there was no difference in the overall survival when comparing the two HERV-W vaccinated groups with each other (Figure 4G). 

## 4. Discussion

In a recent study, we generated a potential cancer vaccine targeting HERV-W which encoded the sequence for the fusogen Syncytin-1 and an assembled HERV-W Gag sequence [18]. Following this study, we were interested in developing a non-fusogenic HERV-W vaccine without compromising the vaccine’s immunogenicity. Therefore, in this present study, we tested two previously reported non-fusogenic HERV-W Env/Syncytin-1 mutants: one mutant containing a neutral amino acid substitution (C > A) in the disulfide bond motif of the Env TU ectodomain, and one mutant with four amino acids (LQMV) inserted into the Env CT, restoring an evolutionary lost cleavage site of the fusion inhibitory R-peptide. Neither of the HERV-W mutants induced fusion, but only the HERV-W_LQMV_ mutant increased Env cell surface expression in HEK293 cells and was clearly expressed on the cell surfaces of T24 cells. Furthermore, mutating the disulfide bond might affect protein folding; therefore, we proceed with the HERV-W_LQMV_ variant. Interestingly, when encoded in an hAd19a/64 vector, the HERV-W_LQMV_ mutant also induced higher levels of Env cell surface expression than the HERV-W_WT_ vaccine in both murine BMDCs and human A549 cells. 

Next, we assessed potential differences in innate immunogenicity between the HERV-W_WT_ and HERV-W_LQMV_ vaccines by measuring the expression of cell surface activation markers and secreted proinflammatory biomarkers of transduced murine BMDCs. Most metrics were similar between the two vaccines, but we observed a reduction in CD40 cell surface expression concomitant with an increase in TNFα in the supernatant of HERV-W_LQMV_ transduced BMDCs. It was not possible to pinpoint a more or less activated phenotype of these transduced BMDCs, but as previously mentioned, the BMDCs confirmed the HERV-W_LQMV_ vaccine-induced increase in Env protein cell surface expression. 

Increased antigen expression has previously been found to increase T-cell responses to adenovirus-vector-encoded antigens [27]. Interestingly, prior studies have also shown that dramatically increased antigen presentation or diminished epitope competition can yield similar modest effects on the responses to dominant epitopes and primarily improve subdominant epitope-specific responses [28,29]. This agrees with what we observed in our study. The vaccination of inbred BALB/c mice with the HERV-W_LQMV_ vaccine resulted in significantly higher CD8^+^ T-cell responses to the HERV-W_LQMV_ vaccine compared to HERV-W_WT_ vaccine at 14 and 28 days after vaccination for the less-responsive epitopes and peptide pools, such as peptide 34 and the SU peptide pool. In contrast, responses to the most immunogenic peptide, peptide 28, were unchanged.

Furthermore, we observed significantly higher humoral IgG responses to the HERV-W_LQMV_ vaccine on day 14 and day 28 after vaccination compared to the HERV-W_WT_ vaccine. Both vaccines improved the overall probability of survival of the mice subjected to distantly injected lung tumours expressing HERV-W Env. However, there was no difference between the two vaccines, likely reflecting similar responses to major epitopes, such as peptide 28. 

To our knowledge, no other study has characterised the immunogenicity of the LQMV mutant of Syncytin-1 nor assessed its expression in multiple cell lines and primary cells. We were consequently surprised by the findings of higher levels of cell surface expression in most cells, as well as higher and broader Env-specific CD8^+^ T-cell responses and antibody responses to the HERV-W_LQMV_ vaccine compared to the HERV-W_WT_ vaccine. While it may seem likely that increased antigen expression can contribute to increased immunogenicity, we cannot be sure that this is the main reason for the observed differences. Further, we have no explanation for why we observe this, nor why this profile was inverted in T24 cells. 

We speculate that the increase in HERV-W_LQMV_ Env cell surface expression via transfection in HEK293 cells and transduced murine BMDCs, as well as the increase in the expression levels of both Env and Gag of transduced A549 cells, could be attributed to changes in protein homeostasis, such as increased production, increased surface accumulation, and decreased breakdown. Alternatively, cell stress can induce increased transactivation of the CMV promoter through which the expression of the HERV-W antigens is determined [30]. The increased CMV promoter activation could increase antigen transcription if the HERV-W_LQMV_ mutant induced more cell stress than the HERV-W_WT_. This could explain why we see higher levels of expression of HERV-W Gag and Env in HERV-W_LQMV_-transduced A549 cells compared to HERV-W_WT_. Furthermore, the increased transcription and expression of antigens caused by cell stress would be expected to affect viability. However, potential differences in cell stress were not reflected in cell viability, which was equal between the groups in both murine BMDCs (Figure 2B) and A549 cells (data not shown).

Oppositely, we did not observe an increased level of Env expression in transfected T24 cells. While A549 and HEK293 cells expressing Syncytin-1 fused modestly, Syncytin-1 expression in T24 cells induced the formation of large, multinucleated cells. Syncytin-1-expressing TE671 cells are also highly fusion-permissive (Figure 2 of [3]). When these cells were transfected with the LQMV mutant in the original study by Bonnaud et al., the Env surface expression of the LQMV mutants was similarly slightly lower than for the wt Syncytin-1 (Figure 1C of [21]). Thus, differences in surface expression of the LQMV mutant may reflect cell-dependent differences in receptor engagement. 

Two fusion receptors for Syncytin-1 have been identified in humans: the sodium-dependent transporters ASCT1 and ASCT2 [3,31]. Mice express ortholog versions of these transporters (murine ASCT1 and ASCT2), which are quite diverse from the human ones. However, early studies showed that pseudo-typed Syncytin-1 virions could infect via the murine ASCT1 or the de-glycosylated version of murine ASCT2 [31]. A more recent study reported the cell–cell fusion of murine B16F10 cells expressing Syncytin-1 [32]. However, both studies indicated that the cell–cell fusion induced by Syncytin-1 in murine cells was less than in human cells. 

We did not observe cell–cell fusions of HERV-W_WT_-transduced murine BMDCs (data not shown) in this study, nor in a similar experimental setup with the transduction of human PBMC-derived dendritic cells (data not shown). This could indicate that cell–cell fusion is highly regulated in antigen-presenting cells. However, in vivo vaccination studies in mice involve many different cell types for which cell–cell fusion induced by Syncytin-1 is possible. Still, cell–cell fusion induced by Syncytin-1 would most certainly be more pronounced in humans. Therefore, we might observe greater differences in cellular and humoral responses between the two HERV-W vaccines if tested in humans. Accordingly, the HERV-W_LQMV_ would not only be a more desired vaccine with respect to fusion-related side effects but it would also have a higher potential to break immunological tolerance in humans. These differences in the mechanism of action in humans versus mice gain further importance when considering that HERV-W is a self-antigen in humans and it is thus likely that there will be a reduced frequency of immune precursors recognising HERV-W with high affinity. Here, it is highly encouraging to observe an enhancement of subdominant antigen T-cell responses by the HERV-W_LQMV_ vaccine, demonstrated by a broadening of the anti-HERV-W Env T-cell response. Indeed, vaccines designed for increased MHC-I- and MHC-II-restricted antigen presentation have demonstrated a wider efficacy gap to non-modified antigens when targeting cancers through their dominant epitopes in tolerant models [33]. Therefore, the HERV-W_LQMV_ vaccine may outcompete the HERV-W_WT_ vaccine in the treatment of human HERV-W-expressing cancers, despite eliciting similar levels of dominant epitope immunogenicity and anti-cancer efficacy against HERV-W-expressing cancers in the highly immunogenic murine system. Additionally, the immune responses to the HERV-W_LQMV_ vaccine would, at the very least, be less sensitive to immune escape.

Whether or not the improvements in HERV-W_LQMV_ immunity will translate into anti-cancer efficacy in humans relies, first of all, on breaking the immunological tolerance to the endogenous (self-antigen) target in the tumour, here HERV-W, without inducing autoimmune reactions to other tissues potentially expressing HERV-W antigens at low levels. Breaking tolerance and avoiding autoimmunity remain major challenges in cancer immunotherapy. Innate and adaptive immune responses to HERV-W in pathological conditions, such as multiple sclerosis and type 1 diabetes, indicate an incomplete tolerance which can be broken in inflamed conditions [34,35,36,37]. Whether a vaccine-induced response can break tolerance in humans and cause autoimmune reactions cannot be answered before tests in humans, but it is encouraging that having multiple sclerosis does not necessarily imply acquiring type 1 diabetes and vice versa.

To test the claim that subtle differences in antigen display and immunogenicity translate into wide differences in a tolerant system, we explored vaccines against a murine endogenous melanoma-associated retrovirus (MelARV). The Env of MelARV is a murine self-antigen overexpressed in many cancer models [38]. In a previous study, we showed that mice were tolerant to DNA plasmids expressing wt MelARV Gag and Env. Still, we could break the immunological tolerance to MelARV through the use of viral vectors [39]. Additionally, via the delivery of a vaccine with immunogenic replication-deficient adenoviral vectors in a prime-boost regimen, in combination with relief of the immunosuppressive activity of the intrinsic Env immunosuppressive domain (ISD) via two point-mutations, we achieved considerably stronger T-cell responses of up to 10% of the CD8^+^ T-cells [25]. These data illustrate that gradually augmenting immunogenicity by improving the delivery vehicle and antigen used allows for an intrinsically tolerant antigen to induce responses of the magnitudes that are typically associated with live viral infections. Furthermore, the MelARV study showed that the adenovirus-vectored vaccine encoding the MelARV Gag and the mutated Env, in combination with the immune checkpoint inhibitor, anti-PD-1 antibody, could eradicate established colorectal tumours in mice [25].

Our current HERV-W vaccines are also encoded in adenoviral vectors, but in contrast to the MelARV study, a mutation of the ISD is unnecessary because the ISD of the HERV-W Env Syncytin-1 is natively non-immunosuppressive [18,40]. Notably, the non-immunosuppressive ISD of Syncytin-1 might help explain the reported spontaneous break of tolerance in humans [34,35,36]. While that presents an obstacle to further improving the immunogenicity of Syncytin-1 through changes to its ISD [25,41], the HERV-W_LQMV_ mutant here represents a fortunate and surprising strategy for increasing the HERV-W vaccine immunogenicity. 

## 5. Conclusions

In this study we generated a non-fusogenic HERV-W cancer vaccine capable of inducing cellular and humoral immune responses without the risk of causing undesired and potentially dangerous vaccinate-induced cell–cell fusions. We found that the HERV-W_LQMV_ vaccine significantly improved survival in mice with HERV-W Env-positive cancers compared to the control vaccine. Relative to the wt HERV-W vaccine, the HERV-W_LQMV_ vaccine increased specific immunogenicity, which enhances the prospects for developing immunotherapies for patients with HERV-W-overexpressing cancers. 

## Data Availability

Data is contained within the article or Supplementary Material. The raw data is available upon reasonable request from the corresponding author.

## Supplementary Materials

The following supporting information can be downloaded at: https://www.mdpi.com/article/10.3390/v15081686/s1.

## Abbreviations

BMDC	Bone marrow-derived dendritic cells
CT	Cytoplasmic domain
Env	Envelope
Gag	Group-specific antigen
hAd19a/64	Human adenovirus type 19a/64
HERV	Human endogenous retrovirus
HERV-W_C>A_	HERV-W Env with C to A mutation, HERV-W Gag
HERV-W_LQMV_	HERV-W Env with LQMV insertion, HERV-W Gag
HERV-W_WT_	HERV-W Env wt, HERV-W Gag
IFU	Infectious units
ISD	Immunosuppressive domain
i.v.	Intravenous
MelARV	Melanoma associated retrovirus
MFI	Geometric mean fluorescent intensity
MHC-I/MHC-II	MHC class I/MHC class II
MOI	Multiplicity of infection
Neg. ctrl	Negative control
Pen/strep	Penicillin-streptomycin
PFA	Paraformaldehyde
P28	Peptide 28
P34	Peptide 34
s.c.	Subcutaneous
SU	Surface subunit
TEM	Transmission electron microscopy
TM	Transmembrane domain
TU	Transmembrane subunit
VLP	Virus-like particle
wt	Wildtype
