# Supplementary material for: The Insertion of an Evolutionary Lost Four-Amino-Acid Cytoplasmic Tail Peptide into a Syncytin-1 Vaccine Increases T- and B-Cell Responses in Mice
Source: Viruses. 2023 Aug 3;15(8):1686. doi: 10.3390/v15081686 (PMC10458386; doi:10.3390/v15081686)
Supplement: Supplementary file 1 [file viruses-15-01686-s001.zip › viruses-2465081-supplementary.pdf]

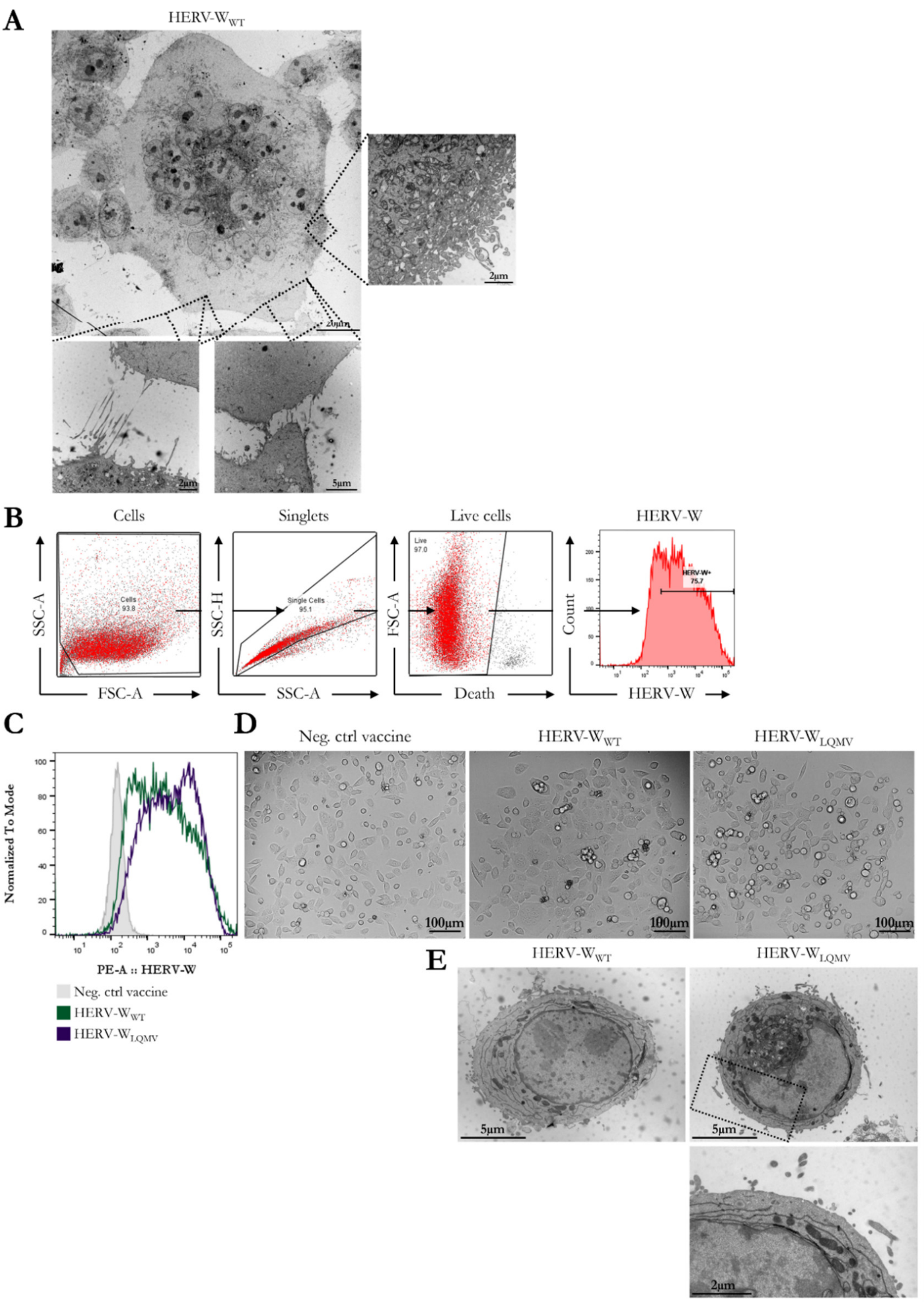

Supplementary Figure S1. (**A**) Transmission electron microscopy (TEM) pictures of a multinucleated T24 mega cell 24h after transfection with HERV-W$_{WT}$ plasmid. Zoom-in to the right shows the advanced surface morphology of the mega cell, and the two bottom pictures show protrusions between the mega cell and the neighbouring cells. (**B**) Flow cytometry gating strategy for HERV-W Env surface expression of transduced A549 cells. (**C**) Histogram overlay of HERV-W Env surface expression of A549 cells transduced with either the two HERV-W vaccines or the empty control vaccine (Neg. ctrl vaccine). (**D**) Light microscopy pictures of A549 cells transduced with one of HERV-W vaccines or the Neg. ctrl vaccine. (**E**) TEM pictures of A549 cells transduced with the HERV-W vaccines. The bottom right picture illustrates a higher magnification of the cell membrane of the picture above.

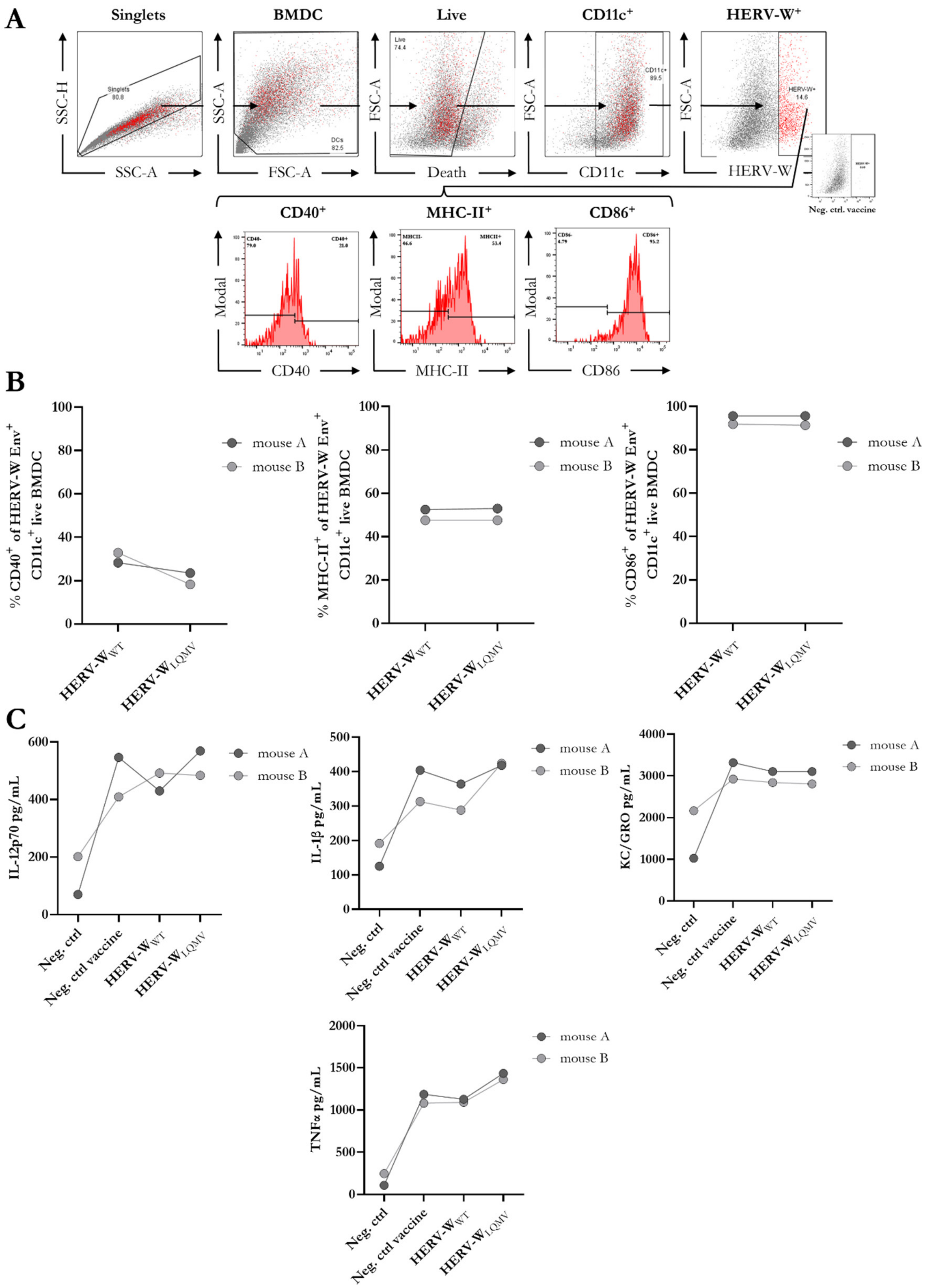

Supplementary Figure S2. (**A**) Flow cytometry gating strategy for transduced BMDCs. Samples transduced with the empty control vaccine (Neg. ctrl vaccine) and fluorescent-minus-one samples were used to set gates for the activation markers CD40, MHC-II, and CD86 as well as the gate for HERV-W Env-expression. (**B**) Percentage of CD40 (left), MHC-II (middle), and CD86 (right) expressing BMDCs out of the live, CD11c+, HERV-W Env+ cells. (**C**) Concentrations of the proinflammatory biomarkers IL-12p70, IL-1β, KC/GRO, and TNFα in the supernatant of BMDCs 24h after transduction. All bullets depict the mean of two technical repeats from each of the two mice.

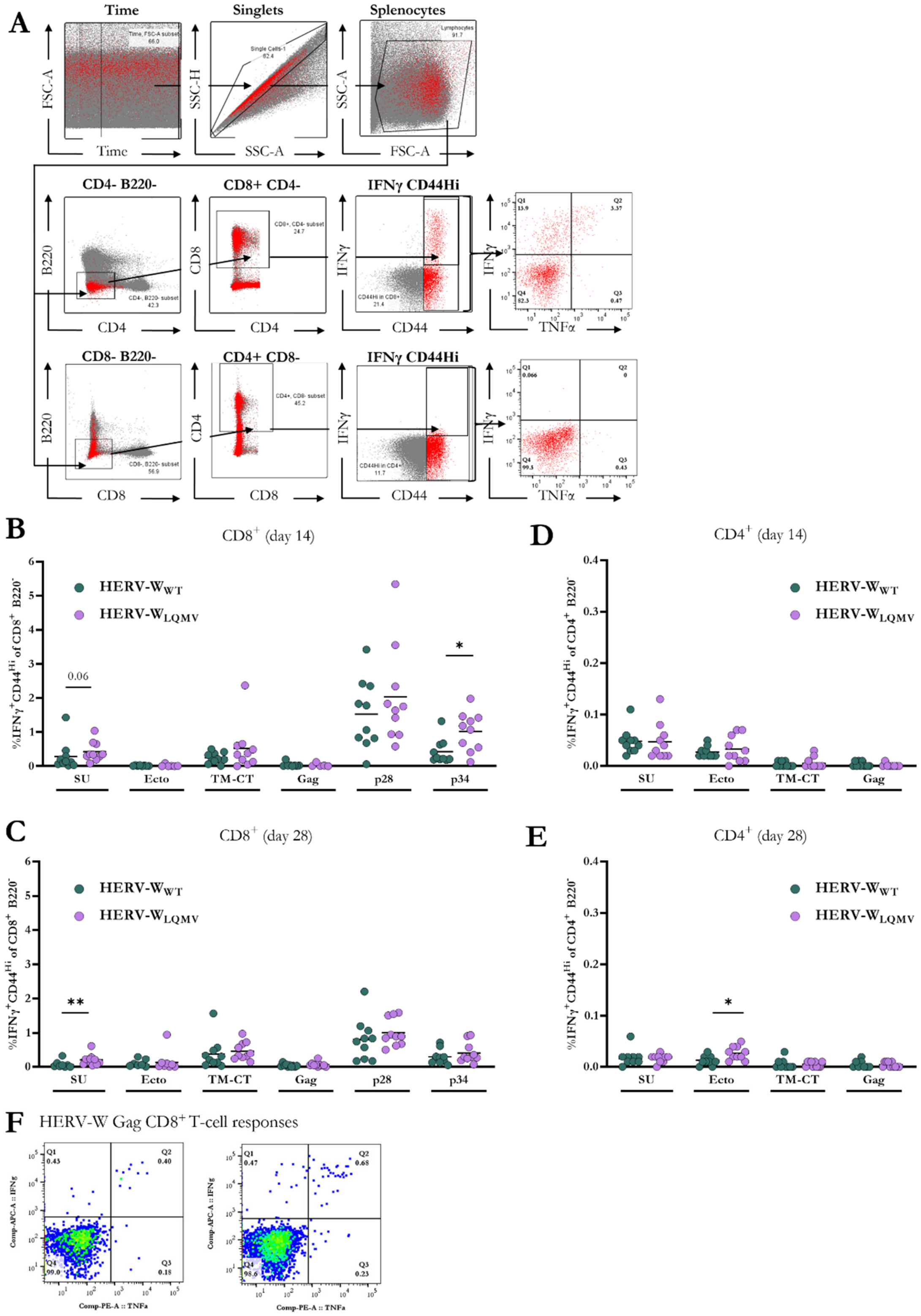

Supplementary Figure S3. (**A**) Flow cytometry gating strategy of intracellular staining of CD8+ and CD4+ T-cell responses to the HERV-W vaccines. Percentage of IFNγ-responding CD8+ T-cells on day 14 (**B**) and day 28 (**C**) after vaccination, or CD4+ T-cells on day 14 (**D**) and day 28 (**E**) after vaccination. Each bullet represents one mouse, and the mean is indicated with a line in bold. Statistical significance between the two vaccine groups is calculated using the non-parametric two-tailed Mann-Whitney test and significance levels are defined as * = $p<0.05$, ** = $p<0.01$. (**F**) The two dot-plots illustrate IFNγ+ CD8+ T-cells from the two mice, one from each group, responding to the HERV-W Gag peptide pool 14 days after immunisation.

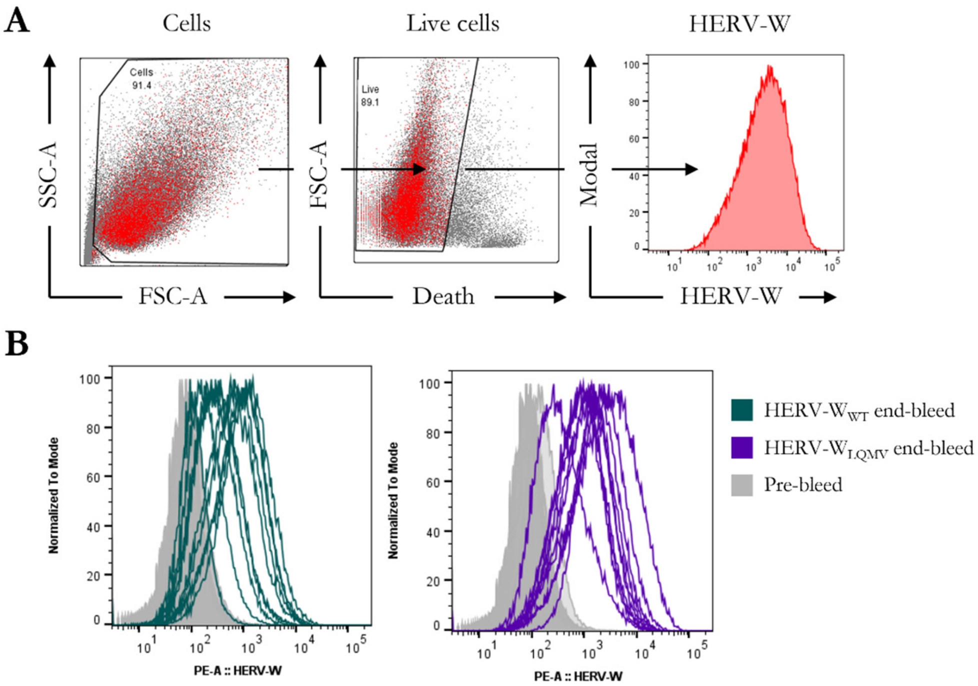

Supplementary Figure S4. (**A**) Gating strategy for flow cytometry of serum IgG binding to HERV-W Env$^+$ RenCa cells. (**B**) Histograms illustrating response differences of serum IgG to HERV-W Env$^+$ RenCa cells between pre-bleed and end-bleed of all the mice vaccinated wit
